# Neurophysiological Measures of Efficacy and Safety for Botulinum Toxin Injection in Facial and Bulbar Muscles: Special Considerations

**DOI:** 10.3390/toxins9110352

**Published:** 2017-10-30

**Authors:** Mohammad Alimohammadi, Anna Rostedt Punga

**Affiliations:** 1 Department of Medical Sciences, Uppsala University, 75185 Uppsala, Sweden; Mohammad.Alimohammadi@medsci.uu.se; 2 Department of Neuroscience, Clinical Neurophysiology, Uppsala University, 75185 Uppsala, Sweden

**Keywords:** botulinum toxin, glabella, facial muscles, CMAP, diffusion

## Abstract

Botulinum toxin (BoNT) injections into facial and bulbar muscles are widely and increasingly used as medical treatments for cervical and facial dystonia, facial hemispasm, correction of facial palsy, hyperhidrosis, as well as cosmetic treatment of glabellar lines associated with grief and anger. Although BoNT treatment is generally considered safe, the diffusion of the toxin to surrounding muscles may result in complications, including difficulties swallowing, in a dose-dependent manner. The sensitivity of clinical examination for detecting adverse events after BoNT treatment is limited. Few reports have highlighted the potential effects on other muscles in the facial area due to the spreading of the toxin. The possibilities of spreading and thus unknown pharmacological BoNT effects in non-targeted muscles emphasise the importance of correct administration of BoNT in terms of dose selection, injection points, and appropriate effect surveillance. In this review article, we will focus on novel objective measures of efficacy and safety regarding BoNT treatment of facial muscles and the reasons why this is important.

## 1. Introduction

Botulinum toxin (BoNT) is a highly potent neurotoxin produced by several species included in the Gram-positive anaerobic Clostridia bacteria family, among others *Clostridium Botulinum*, where each serotype is produced by a particular strain of *Clostridium Botulinum* [1]. In addition to the seven previously identified serotypes (A-G) of BoNT, another serotype was recently identified as BoNT/X [2], and the first botulinum-like toxin outside the Clostridia family has been described [3]. 

In the clinical setting, the use of BoNTs is restricted to the type A and B serotypes, of which the type A serotype is most widely used in small quantities as a treatment in aesthetic and medical indications, most of which are characterised by increased muscle activity (reviewed in [4]).

The toxin acts by blocking the pre-synaptic fusion of acetylcholine-containing vesicles, and in this way inhibits the neuromuscular transmission to nicotinic acetylcholine receptors and thus muscle activation [1]. The heavy chain of BoNT mediates the binding to nerve terminals and the membrane translocation of the light chains into the cytosol, where the substrates of BoNT, namely the three SNARE proteins (VAMP, SNAP25, and syntaxin), are localised. Recently, it was described that the heat shock protein Hsp90 is involved in the entry of clostridial neurotoxins into the cytosol of nerve terminals [5,6,7]. The subsequent effect of BoNT is mediated by the inhibition of acetylcholine release from the motor nerve terminal into the neuromuscular junction [8]. Growing evidence indicates that BoNTs also inhibits the action of other neurotransmitters such as substance P and calcitonin gene-related peptide, which mediate pain and neurogenic inflammation [9]. Consequently, one can expect that the clinical use of BoNTs will increase in future medical applications. 

## 2. Indications and Special Considerations of Safety Regarding BoNT Applications in Facial and Bulbar Muscles

### 2.1. General Indications and Mode of Action

A wide range of medical conditions involve treatments with BoNT to counteract muscular hyperactivity/tension in the facial muscles innervated mainly by the facial nerve and bulbar muscles, which are in turn innervated by cranial nerves of the lower brain stem. The medical conditions include a wide variety of neurological disorders, such as spasmodic torticollis, cervical and facial dystonia, blepharospam, facial hemispasm, and spasticity, as well as other indications such as cluster headache/migraine [10,11,12]. The targets for aesthetic treatment are broad and can be exemplified by the treatment of glabellar rhytids caused by tension in the procerus and corrugator muscle complex; horizontal forehead lines caused by tension in the frontal muscle; periorbital rhytids caused by tension of the orbicularis oculi muscle; perioral vertical rhytids (smokers line) caused by tension in orbicularis oris muscle; and gummy smile caused by tension in the lip levator. 

In 2002, the Food and Drug Administration (FDA) approved treatment with BoNT for the temporary correction of glabellar frown lines and since then, treatment has been extended to hyperdynamic rhytids of both the upper and lower face. Treatment indications have expanded to include targeted treatment of muscles in both the mid and lower face, including the temporary correction of unwanted lower face hyperdynamic rhytids and facial contouring [13]. 

### 2.2. Spreading of the Toxin’s Effect Can Give Rise to Undesired Side Effects

Despite the general assumption that botulinum toxin is safe, its widespread use and the ever-expanding indications raise issues of safety due to its neurotoxicity. There is also the risk of unwanted adverse effects due to spreading of the toxin [14]. Intriguingly, recent findings challenge the established view that BoNT trafficking is restricted merely to the neuromuscular junction, and instead suggest more distant trafficking mechanisms since BoNT/A, including internalisation of the toxin by spinal cord motor neurons and fast axonal retrograde transportation. Post-marketing data with BoNT products indicate that the toxin’s effect is sometimes observed beyond the site of local injection. The most serious adverse events, reported hours to weeks after treatment with BoNT, include death, sometimes associated with anaphylaxis, dysphagia, respiratory insufficiency, and generalised muscle weakness [15,16]. It should be clarified, however, that these rare systemic events were only seen at excessively high dosages [17] or in patients with underlying medical conditions that were predisposed to the complication, and thus the BoNT injection itself did not cause the death of these patients [14,18]. Severe symptoms have been reported hours to weeks after injection and most of the events are associated with BoNT injection for cervical dystonia and limb spasticity. 

A systematic review of clinical studies to evaluate the safety of botulinum toxin A in aesthetic treatments between 2000 and 2012, including 35 papers with a total of 8787 subjects, was performed in 2014 [19]. Treatment-related adverse events were blepharoptosis (2.5%), brow ptosis (3.1%), and eye sensory disorders (3%) in the upper face and lip asymmetries and imbalances in the lower face (6.9%). All of these events resolved spontaneously. These adverse events may be dose-dependent and were attributed to local diffusion of BoNT into adjacent areas, such as neck extensor myopathy and dysphagia, especially in the treatment of neck and bulbar muscles in torticollis [20]. Thorough knowledge of the anatomy and physiology of the treated muscles as well as of the pharmacology of the drug is imperative to avoid these serious adverse events, which can occur hours to weeks after the initial therapy. 

## 3. Muscle Anatomy and Physiology in the Facial and Bulbar Area

### 3.1. Facial Muscles are Extra Sensitive to Denervation

One important factor to consider for intramuscular BoNT injection is the various responses of different muscles to chemical denervation with subsequent reinnervation [21,22]. Muscles can be grouped into two classes, depending on their embryonic development and their response to denervation. So-called ‘delayed synapsing’ (DeSyn) muscles exhibit less compact acetylcholine receptor (AChR) clusters and a substantial level of extrasynaptic AChR clusters, whereas ‘fast synapsing’ (FaSyn) muscles accumulate compact focal clusters with low levels of AChR along the muscle fibre [23]. Interestingly, presynaptic blockade of neuromuscular transmission with BoNT causes extensive motor nerve sprouting, i.e., reinnervation response, in DeSyn but not in FaSyn muscles [23]. One explanation for this differential response to blocked neuromuscular transmission could be that adult skeletal muscles differ in their endogenous levels of the important receptor muscle specific tyrosine kinase (MuSK). In turn, this difference correlates with the ability to form ectopic AChR clusters and thereby also reinnervate [24]. Facial and bulbar muscles, including the omohyoid and masseter muscles, harbour low levels of MuSK and these muscles are more vulnerable to denervation upon blocked neuromuscular transmission [25]. Thus, excessive doses of BoNT may render these muscles atrophic, and unwanted side effects may arise that limit normal facial muscle functions such as chewing (masseter).

### 3.2. Specific Target Treatment in Facial Muscles

**Figure 1:**
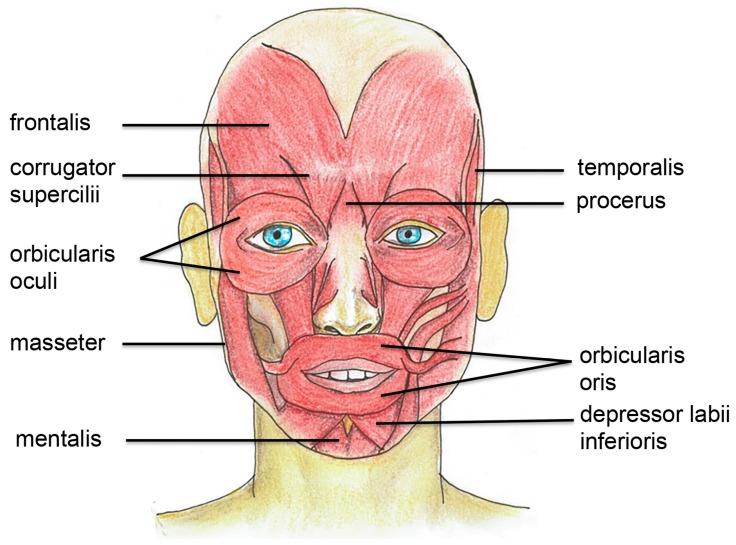
Schematic illustration of the facial muscles that are commonly injected with botulinum toxin both for aesthetic and medical purposes.

BoNT is commonly used to treat both glabellar lines and forehead lines, through intramuscular injections in the corrugator supercilii, procerus, and frontalis muscles [26,27]; however, the indications are constantly increasing. BoNTA was first reported to be effective cosmetically once shown to reduce the appearance of glabellar folds [26]. A schematic overview of the facial muscles involved for the indications below is provided in Figure 1.

#### 3.2.1. Glabellar Muscle Complex

The glabellar muscle complex attracts attention from both patients and their observers due to its prominent location centrally on the face. Rhytids in this region are most often dynamic in nature and range from fine lines to deep furrows as a result of the pulling of the skin by the underlying muscle [28]. Detailed anatomical studies have revealed that the corrugator muscle thickness varies significantly depending on the location being measured (approximately 2–3 mm), whereas the procerus is fairly consistent in thickness (≤1 mm) throughout its course [28]. In the medial canthal area, the thin frontalis muscle is encountered superficially to the corrugator supercilii muscle located more deeply, whereas the corrugator becomes confluent with the procerus medially [28]. Detailed knowledge of the exact anatomical relationships between the main muscles to be injected in the upper part of the face is very important to avoid the development of adverse effects. In particular, upper eyelid ptosis may be caused by improper needle placement as a result of the migration of the BoNT through the orbital septum to the levator palpebrae superioris muscle. Hyperfunctional lines of the glabellar muscles can be treated by injection of BoNT in the procerus and corrugator muscle complex. The results are relaxation of transverse and vertical rhytids located between the eyebrows in the glabellar region. This was the first FDA-approved cosmetic use of BoNT and the risk for possible diffusion that causes eyelid ptosis is low, approximately 2.5% [19]. In addition, as the procerus and corrugators muscles function as depressors of eyebrows, the treatment of glabella also results in the elevation of the eyebrows.

#### 3.2.2. Frontalis Muscle and Horizontal Rhytids

Horizontal rhytids of the forehead are treated by injection of BoNT into the frontalis muscles at approximately six to 12 injection points, and the subsequent partial paralysis of the frontalis muscle results in abolishment of the forehead rhytids [29]. Also in the case of frontalis muscle treatment, there is a risk of eyebrow ptosis. Directly superior to the upper part of the nose, the frontalis fibres are continuous for a variable distance before an aponeurosis occupies the space between the two bilateral muscle bellies. The location of divergence of the two muscle bellies is known as the dehiscence point or midline attenuation point. It has been noted from anatomical studies that approximately one third of females have a contiguous frontalis muscle to 6 cm above the orbits [30]. These individual differences of the frontalis muscle may play an important role in the diffusion properties of BoNT. 

#### 3.2.3. Orbicularis Oculi Muscle 

Periorbital lines, also known as crow’s feet, are caused by tension in the orbicularis oculi muscle. This results in radiating rhytids in the lateral orbital rim when the patient squints or smiles. By injection of low doses of BoNT in the orbicularis oculi muscle at one to three injection points, the crow’s feet can be abolished [31]. 

#### 3.2.4. Brow Lift

Brow lift by chemical denervation with BoNT trough treatment of the glabellar complex and the orbicularis oculi has favourable and long-lasting aesthetic results with minimal morbidity. Nevertheless, brow lifts may have an unpredictable cosmetic outcome with asymmetry and usually a touch-up treatment is needed to correct asymmetries [32]. 

#### 3.2.5. Orbicularis Oris, Mentalis Muscle, and Lip Levator

Vertical lip rhytids (also known as “smoker’s lines”) occur due to tension of the orbicularis oris muscle and appear both on the upper and lower lip. These rhytids can be treated by direct injection of BoNT into the orbicularis oris at six injection points. There is a high risk for lip asymmetry and possible speech or drinking impairment, likely due to relaxation of the orbicularis oris. 

Marionette lines or melomental rhytids appear in the lateral aspect of the perioral region due to the contraction of orbicularis oris. These vertical lines may give the impression of sadness and can be treated in the same way as treatment of smoker’s lines. However, lateral injection sites are selected.

Mentalis crease is tension in the mentalis muscle that results in a sagittal rhytid/crease on the chin. This is predominantly seen in male subjects. The condition can be perceived as a high level of psychological discomfort in some individuals and can be treated with BoNT injected into the mental muscle.

Gummy smile is seen in some subjects and is due to anatomical variation; nevertheless, the condition can be treated by chemical denervation of the lip levator [33]. This treatment is cost-effective and minimally invasive in comparison with surgery. 

#### 3.2.6. Masseter Hypertrophy

The masseter is the most powerful masticatory muscle, responsible for jaw closing and thus chewing. Due to its more prominent thickness in Asians than in Caucasians, Asian women in particular are interested in reducing the masseter volume in order to improve the lower facial contour [34,35]. BoNT injection has been widely performed in recent years to treat masseteric hypertrophy and recently also for chronic masticatory myofascial pain [36]. Due to its thick muscle volume, the masseter usually requires a moderate dose of BoNT in order to relax. However, repetitive treatments can result in muscular atrophy, with subsequent undesired adverse events such as chewing difficulties. A recent prospective case series looked at patients with chronic masticatory myofascial pain (MMP) treated with injection of BoNT into the bilateral temporalis and masseter muscles [37]. Since pain decreased significantly, this study suggested BoNT as a safe and effective treatment for chronic MMP.

## 4. Neurophysiological Measures of BoNT Effect in Facial Muscles: Important Objective Parameters and Guiding Techniques

### 4.1. Application of Neurophysiological Measures

Neurophysiological techniques have been used for many decades in order to optimise BoNT treatment of cervical dystonia and torticollis. There is class I evidence that in treatment-naive patients, improvements in cervical dystonia and pain are greater if muscles are selected based on a combination of clinical examination and electromyography guided injections [38,39]. In dermatologic and cosmetic practices, on the other hand, clinical rating scales merely include visual assessment of wrinkle depth as well as maximal muscle contraction. These visual scales are commonly used to guide treatment and as measures of the efficacy of BoNT in facial muscles [40,41]. Although cosmetically useful, these scales are not considered sensitive enough to detect subtle differences in effect between various formulations or doses of BoNT, nor to determine the exact onset of action [42,43,44]. Because the desired pharmacological effect of BoNT is muscle paralysis, neurophysiological parameters are crucial to quantify the functional effect on the muscles being injected. Older neurophysiological studies that compared different BoNT formulations mainly measured the collective muscle response upon motor nerve stimulations, also known as the compound motor action potential (CMAP), mainly in the foot extensor muscle [45,46,47]. 

### 4.2. CMAP and EMG as Indicators of BoNT Effect

Mostly based on studies of CMAP, which show a dose-response reduction upon intramuscular BoNT injection in the extensor digitorum brevis (EDB) muscle of the foot, there is a general consensus that the CMAP measure provides a reliable evaluation of neuromuscular function [48,49]. More recently, randomised prospective double-blind studies have obtained also evidence for the CMAP as an important objective measure of BoNT efficacy in the small glabellar muscles.

The first study found that reduction of the CMAP amplitude measured over the corrugator supercilii correlates well with intramuscular dose of onabotulinumtoxin A (Vistabel^®^) in the same muscle [50]. Further, electromyography (EMG) measurements revealed the onset of muscle paralysis after two weeks and effects (denervation) lasting as long as 24 weeks after injection in individuals receiving a high dose of onabotulinumtoxin [50]. Importantly, this study supports a novel neurophysiological strategy for effect evaluation of BoNT in glabellar muscles, since both CMAP and EMG parameters correlated with BoNT dose. Another study recently used neurophysiological parameters in the application of BoNT in the periorbital area for the treatment of crow’s feet [51].

EMG was also applied in the BoNT treatment of masseteric hypertrophy. EMG results from maximal jaw muscle contraction showed a greater decrease in the patient group who received a dose of 25U compared to 35U at all follow-up time points after injection; however, there was no statistically significant difference between the two groups [52]. 

### 4.3. Ultrastructural Muscle Changes Indicating Denervation after BoNT Injection

EMG and CMAP indirectly show the function of the motor nerve and its innervated muscle. A few studies have analysed the ultrastructural changes in muscle after BoNT injection, and these findings correspond with the time scheme observed with electrophysiological outcome measures. Ma F. et al. found significantly distorted arrangements of muscle fibres in the masseter muscle, similar to the changes observed after denervation, at six months after BoNT injection in patients with masseteric hypertrophy [53]. The findings of shorter sarcomeres and abnormal mitochondrial structure at six months improved until 12 months after BoNT injection, but did not entirely normalise [53].

Dengis et al. reported that botulinum toxin affected the proprioceptive feedback of the extraocular muscles over a long-term period [54]. Although the mechanism by which BoNT alters proprioceptive feedback is not established, proprioceptive changes can occur if the toxin induces structural changes of myotendinous nerve endings. Recent studies in cats revealed that BoNT injection of the extraocular muscles changed the ultrastructure of palisade endings in the myotendonous area, which might cause abnormal proprioceptional input [55]. Since these alterations can influence the proprioceptive abilities of the extraocular muscles, possible proprioceptive dysfunction after BoNT injection into these muscles for the treatment of strabismus should be considered. Further, tearing and dehiscence in the myelin sheath, axonal dispersion, degeneration in the Schwann cell cytoplasm, and degenerative changes were reported 12 weeks after injection of BoNT into the anterior auricular muscle of rabbits [56].

### 4.4. Terminal Sprouting Causes Reinnervation

The return of muscle function after BoNT injection is caused by the sprouting of axonal collaterals from the presynaptic nerve endings at the neuromuscular junctions of the paralyzed muscles [57]. Nerve sprouting after BoNT treatment results in a significant increase in new acetylcholine receptors on the treated muscle compared to normal. These newly formed acetylcholine receptors are in locations distinct from those of the original, paralyzed neuromuscular junctions [58]. Together, these observations suggest that terminal nerve sprouts elicited by BoNT grow robustly outside the synapse area on the muscle fibre surface and can be measured as early as three days after BoNT injection [59]. CMAP studies demonstrate the return of 20% of normal activity in patients as soon as seven days after BoNT injection in the EDB muscle [60]. Interestingly, in the glabellar muscles a substantial reduction in the CMAP parameter was still seen at six months after treatment, with values reduced to approximately 60% of the baseline amplitude in the labelled volume and to a numerically greater reduction of approximately 50% in the twofold volume group [61]. The reduction in CMAP was greater in the group who received the twofold injection volume at every time point, including time to the onset of effect and duration. This suggests that the volume possibly plays a role in the diffusion to involve more neuromuscular junctions and thus increase the duration of action of BoNT.

## 5. Considerations to “Split Face” Design: Spread of BoNT in the Facial and Bulbar Area

Documented possibilities of still unknown pharmacological BoNT effects emphasise the importance of correct administration of the toxin in terms of injection points, dose selection, and appropriate effect surveillance. A considerable number of clinical trials comparing different forms of BoNT in the cosmetic industry are designed in a split-face manner, which does not take into account the regional diffusion of the toxin to the contralateral side of the face [62,63,64]. There are no measurable circulating biomarkers to detect this spread except for the mentioned parameters of denervation activity of EMG, implicating an active chemical denervation process in injected and adjacent muscles, in addition to reduced muscle response (CMAP) and disturbed neuromuscular transmission measured by single-fibre EMG. 

### 5.1. Possible Diffusion and Migration Mechanisms of BoNT

Diffusion is the movement of the toxin beyond the immediate injection site [65,66] by Brownian motion, and is determined by the concentration gradient and the BoNT molecular size. Migration, on the other hand, is the distant hematogenous and neuroaxonal transport of BoNT from the muscle and is related to systemic side effects [67,68]. Surprisingly, few data are available on convection and diffusion in human muscle [65]. Nevertheless, several animal studies demonstrate that BoNT moves into adjacent muscles when injected in limb muscles [69,70] as well as in bulbar muscles [71]. Bulbar muscles are innervated by cranial nerves IX, X, XI, and XII, and include the tongue (m glossus), pharyngeal, and laryngeal muscles as well as the sternomastoid and upper trapezius muscles. Diffusion of BoNT both within the injected muscle as well as to neighbouring muscles has previously been well-documented in isolated muscles [72], irrespective of whether the muscles are separated by fasciae. Further, it is well known that BoNT passes through the muscle fascia easily, even at subclinical doses [73], but that the diffusion to surrounding muscles at least in the bulbar/neck area can be prevented to some extent by using lower BoNT doses [71]. Despite these known diffusion possibilities both in a distal-proximal and contralateral spread of the toxin in the facial and bulbar region, comparisons of two different BoNT subtypes often apply the so called “split-face” design. 

### 5.2. Glabellar and Frontalis Muscle Area

Following injections of the glabellar muscles for the indication glabellar lines, limited diffusion is known to occur to the surrounding extraocular muscle orbicularis oculi. This diffusion can be detected as slightly disturbed neuromuscular transmission (jitter) with single-fibre electromyography [50]. Diffusion has also been reported to contralateral facial muscles following unilateral BoNT application [74]. One recent study aimed at further characterizing the contralateral diffusion of BoNT through unilateral application of onabotulinumtoxin A. A randomised, double blind study was conducted in which five healthy women (33–52 years) were treated with different doses of onabotulinum toxin unilaterally in the corrugator muscle [75]. Parameters of efficacy and diffusion (CMAP, EMG, and jitter analysis) in both glabellar and frontalis muscles were assessed at baseline, two, and four weeks following BoNT injection. Apart from the expected reduction of the CMAP in the treated ipsilateral glabellar muscles, contralateral CMAP reduction was observed in three out of five subjects [75]. This confirmation of the regional diffusion of BoNT in facial muscle applications raises questions about the reliability of split-face models in BoNT studies as well as the possibility of remote spread from the site of injection, even to arm muscles [76]. 

### 5.3. Orbicularis Oculi

One study examined patients treated with BoNT for hemifacial spasm and blepharospasm in the orbicularis oculi muscle and analysed the CMAP of the orbicularis oris muscle [20]. These findings were further supported by a case report of a patient who received a low dose BoNT injection in the orbicularis oculi for blepharospasm and developed hemifacial paralysis with denervation activity seen on EMG in the orbicularis oculi muscle [75]. Since both the orbicularis oris and orbicularis oculi muscles are innervated by the facial nerve, the decline of CMAP in both these muscles suggests spread by axonal diffusion but possibly also by local diffusion. Intriguingly, another study by Lorenzano et al. did not find any clinical or neurophysiological evidence of BoNT by judging the CMAP amplitude in the orbicularis oris muscle on the same side as the treated orbicularis oculi muscle [77]. Nevertheless, minor signs of diffusion, such as increased spontaneous activity on EMG that indicates denervation, is not to be expected in the CMAP measure in circular muscles such as the orbicularis oculi or orbicularis oris muscles due to technical difficulties. 

In conclusion, although treatment with BoNT is considered safe, there are several adverse events that can occur when injecting facial and bulbar muscles, and we have highlighted them in this report. In particular, the knowledge on adverse events due to diffusion to contralateral muscles is limited. We have recently shown that the measurement of objective neurophysiological parameters, including CMAP and EMG, can be used to detect subclinical effects of BoNT. We consider that these types of measurements are more reliable and could be used as outcome measures in randomised clinical trials of BoNT to evaluate both the effects and adverse events of BoNT treatment. Importantly, the issue of BoNT diffusion in the facial area questions the reliability of split-face studies.
